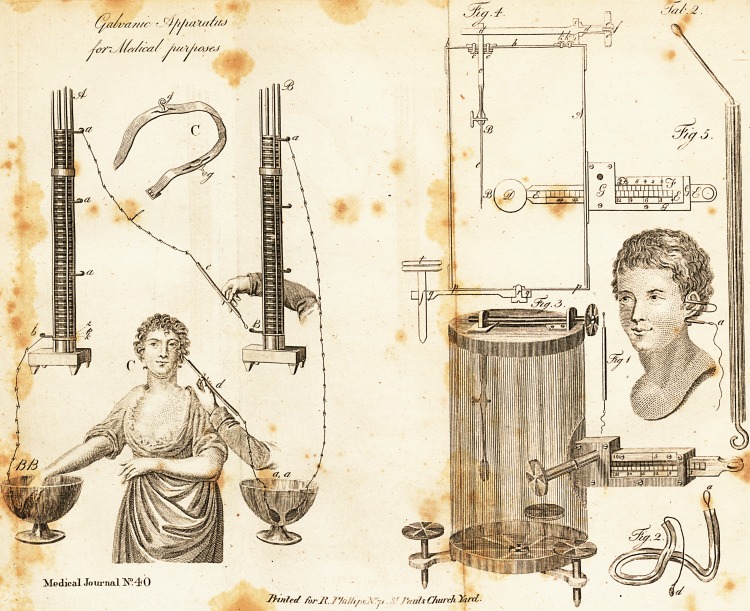# On Galvanism and Its Medical Application

**Published:** 1802-06

**Authors:** 


### *On* Galvanism *and its Medical Application; by Dr.* Bischoff,*

[With Engravings.]

THE difcovery of the Galvanic ftimulus, which has of late been the fubject of fo many curious refearches to Naturalifts, was prefaged by Valli, Hufeland, and Reil, to prove of the utmoft importance and utility to therapeutics from the great effect it fhowed on organifm; but particularly fince the time that Mr. Volta fucceeded in producing the effects of the Galvanic power in a confiderable degree by the conftruction of the remarkable apparatus, which now generally bears the name of Volta's pile or column, we may expect to reap the moft extenfive advantage

* The author has made this interefting inquiry the fubject of his Inaugural Differtation, entitled, *De ufu Galvanismi in arte Medica, Jenae MDCCCI, c. tab. aen.* in which he has premifed a brief furvey of the progrefs made in the knowledge of Galvanifm, and of which we fuppofe the following paper to be for the moft part extracted tage from the medical application of fo powerful and penetrating a ftimulus. The conftruction of that apparatus is founded on certain laws which the Galvanic agens is found to obey, according to the ingenious refearches of Volta and Ritter, who difcovered that its effects arife from the contact of three heterogeneous bodies, a fluid or moift one and two folid ones, to the laft of which belong the metals, ores, coal and molybdena, and to the fluid bodies particularly thofe which are the moft faturated with oxygen. The column confifts of round metallic plates, which reprefent the Galvanic chain, with which the fluid (diluted muriatic acid) is brought into contact by means of round pieces of pafteboard, foaked with the acid fluid. When thefe fingle Galvanic chains are placed one upon another in a greater or lefs number according to the effect which is intended to be produced, the apparatus appears as a column, which is put between four glafs pillars both for fecuring it properly and for infulating it; and it is kept together above and below by means of a cafe made of wood or metal. The whole is infulated by placing under it fome glafs plates; inftead of which I found it more convenient to place the column on a pedeftal, infulated by four glafs feet. When fuch a column is conftructed, according to Volta, in the following féries, filver, zinc, pafteboard; filver, zinc, &c. the metallic plates, which happen to be at the two ends of the column, ought properly not to be regarded as active, becaufe it is found that the two heterogeneous metals act only upon each other through the medium of a moift body. Hence it appears, that the phenomena produced by the two ends of the column, which are called poles, are not to be afcribed to the metallic plates ending the pile, but to thofe which are between them; and in a column therefore thus conftructed the phenomena appearing at the laft filver plate, are properly owing to the zinc placed under it, or to the zinc pole; and, vice verfa, the phenomena produced at the laft zinc plate belong accordingly to the filver pole, for thus the two ends of the column are named, of which the filver pole fhows pofitive and the zinc pole negative electricity. Thefe poles manifeftly differ in the quantitive as well as qualitive action they refpectively exert on organifm, a circumftance which I have not only witneffed in ten or twelve patients that fell under my care, but alfo found by frequent experiments I had otherwife an opportunity of making. The difference in the refpective action of the poles appears to take place,

I. *In the general excitement and in the affection of common sensation.*—The action of the two poles however feems to differ more in quantity than in quality; for though the fenfation tion produced on touching the zinc pole rather refembles a fhock, the fenfation at the filver pole confifts in a very fenfible ofcillation of the nerves, which extends itfelf along their branches, yet the difference feems merely to depend on the degree of irritation, and confequently its quantitive, becaufe I think I have perceived the laft fenfation even at the zinc pole, when the battery acted in a lefs degree. But it remains notwithftanding problematical, whence this quantitive difference arifes.

II. *In the fymptoms of the general irritation of organism produced by the topical irritation of fingle organs, viz.*

1. *In the affection of the organ of tafte.*—It has been repeatedly obferved by the above patients, and in the experiments which I made on myfelf, that on bringing the tongue into contact with the zinc pole, a remarkable fourifh tafte is perceived, which on the application of the tongue to the filver or copper pole is found to be more faline-alkaline. It is likewife remarked, that the affection of the organ of tafte is not only more intenfe, but alfo more lafting at the zinc pole than at the filver or copper pole.

2. *In the affection of the organ of fight.*—On combining the eye with the pofitive pole, the colour of the lightning that is thus produced in the eye will appear light blue or greenifh, whereas it is fiery or reddifh at the eye being brought in contact with the negative pole; but the intenfity of thefe colours depends on the ftrength of the battery. This production of light and colour in the eyes feems, however, to be nothing but a fymptom of general incitation, on account of its taking place without a topical irritation of the organ of fight, a phenomenon frequently obferved in my experiments; for whenever I placed myfelf within the courfe of the Galvanic chain by my hands, I perceived a faint glance and ofcillation in my eyes, and alfo feveral of my patients made the fame remark, particularly on the firft time of its application, when they were not accuftomed to the ftimulus; the objects appeared to them lighter and clearer than ufual.

3. *In the affection of the organ of hearing.*—There feems only to exift a quantitive difference in the action of the poles on the organ of hearing; and the fenfation produced in the ear by the application of Galvanic fluid is much more intenfe and of a longer duration at the zinc pole than at the filver pole.

Notwithftanding this difference in the refpective action of the two poles, the phenomena produced by both poles are evidently fymptoms of irritation, though they be not attended with an increafe of the pulfe, of animal heat, and of the fecretions and excretions. It is to be remarked, that the fenfibility of the patients, particularly in the beginning, increafes by degrees during the application of Galvanifm, and that, confequently, at the fame degree of the Galvanic action without any direct increafe of ftimulus, the affections of the fenfes as well as the contractions of the mufcles become more violent. The phenomena, therefore, which the Galvanic ftimulus produces on the organic body, naturally lead us to conclude, in what difeafes it may be fuccefsfully employed. In all affections, therefore, where the organs are deficient in a proper degree of incitation, either from direct or indirect debility, confequently in amaurofis, paralyfis linguae, either originating in the nervus lingualis and fublingualis, whereby the tafte is affected; or arifing from the nervi laryngei, whereby the organ of fpeech fuffers; farther, in that fpecies of deafnefs which originates in a paralyfis of the acouftic nerve; in all cafes of paralyfes of fingle limbs, chronic rheumatifms, ifchiatic, and in gouty complaints, the application of the Galvanic power may prove of the greateft effect. With this view I have employed Galvanifm in feveral difeafes, and, I may venture to fay, with confiderable fuccefs; but before I communicate the hiftory of thefe cafes, I beg leave to premife fome remarks on the battery itfelf, and on my manner of applying it. My apparatus (fee Tab. I. fig. A. A B.) confifts of a tin plate with its margins bent upwards, which is infulated by four glafs feet; on the middle of this plate is a fquare pedeftal, likewife a tin plate, of the fize of a cube of the diameter of one of the largeft metallic plates ufed for the pile; on the middle of each of the four fides of this pedeftal is foldered a tin tube, receiving the four glafs pillars, between which the pile is to be conftructed: Thefe glafs pillars are kept together above by means of a metallic or wooden hoop. The whole pedeftal is varnifhed to prevent its rufting. The zinc plates are of the fize of a crown piece, and double the thicknefs: Inftead of filver I ufe copper-plates; and the feries in which I conftruct the pile, is copper, pafteboard, zinc, alternately, fo that the copper pole in my battery is at its lower end. In order to prevent the fluid that happens to be expreffed from the pafteboard getting between the two metallic plates, I let the copper-plates be made a little larger than the zinc plates, fo that they project about two or three lines beyond them; for when fome of the fluid is preffed out of the pafteboard that is above the copper-plate, it cannot get between the zinc and copper-plates, but dropping down on the next copper-plate it is abforbed by the pafteboard above it. The fluid, thus expreffed, is collected on the loweft plate of copper, where it can be from time to time eafily taken away by means of a fyringe, which I always make ufe of to wet the pafteboard, pafteboard, if the battery is not frefh conftructed. I think it however more convenient, on account of the trouble which it requires to clean the plates after they have been ufed feveral days fucceffively, to conftruct the pile frefh every day. By this means the efficacy of the battery will not be prevented by the oxydation of the metals, which takes place by the continued ufe of the pile. In order to adapt the ftrength of the battrey, which confifts of one hundred plates, to the ftate of incitability of the different patients, I got two additional hooks (a a) made in the middle of the pile, on which the conducting chain may be applied according to the degree of action requifite: And to determine the proper degree of action more accurately I have propofed a *Galvanometer*, the defcription of which is given at the end of this paper. As the pile might be eafily overturned by the ftarting of the patients, I place two bowls (α α β β) filled with a folution of common falt, at fome diftance into which the conducting chains of the two poles are immerfed, and from thence, by means of other chains, the application to the affected organ is made.* The manner in which it was performed will appear from the following Cafes.

*Case* I. A man, forty years of age, whofe fituation in life expofed him to frequent colds, was on a fudden affected with a dimnefs of fight, which, in the courfe of two years and a half, terminated in complete amaurofis of fuch a degree that he could hardly diftinguifh the largeft objects, which appeared to him as diftant fhades. I began my experiments with feventy ftrata of plates, and conducted the chain of the negative pole partly to the region of the nervus fubcutaneus malae, partly to the nervus frontalis, fupraorbitalis, infraorbitalis, to the eyelids, and at laft to the fclerotica. Having thus continued for three or four days, without the expected fuccefs, I conftructed two batteries, each of fixty ftrata, in the following feries— copper, pafteboard, zinc, copper, pafteboard, zinc, &c. (Tab. I. A. K. p. z.). Having combined the zinc pole of one battery with the copper pole of the other by means of a wire, I conducted the zinc pole and copper pole into the two bowls filled with falt water, and ordered the patient to immerfe his hand into that veffel which contained the conductor of the copper pole, while I brought the zinc pole in contact with the eye of

* On this mode of conducting the Galvanic action into a fluid, Dr. Froriep of Jena built his propofal in his Inaugural Differtation, *Diss. de methodo neonatis afphycticis fuccurendi*; —to ufe Galvanifm as a means of vivifying infants in the *asphyxia neonatorum*, by conducting a chain from one of the poles into a bath, in which the child is laid, and another chain from the oppofite pole to the child itfelf.

of the patient by means of an insulated probe, to which the conductor from the other vessel had been fastened. The patient saw frequent lightning, perceived a burning sensation, and had from time to time strong muscular contractions at the places that were touched. When I had thus continued for above five weeks, twice a day, and increased by degrees the shocks given at each time round the eye from 100 to 200, and those applied to the sclerotica from 8 to 16, and not finding any considerable effect, I added thirty strata of plates to each battery, by which means the lightning became more intense. When I had applied this battery for about fifteen days, I had the satisfaction to be assured by my patient, that the lightning appeared to him much increased, and to extend over the whole eye; and that he perceived, three or four hours subsequent to the appplication, a particular light in the eye: The patient could likewise, since that time, distinguish the reddish lightning of the negative pole, and the bluish colour of the positive, which I considered as a step towards recovery. Induced by these favourable symptoms, I continued the Galvanism, in the application of which, however, I made a remarkable alteration, for the purpose of affecting a more perfect current of the Galvanic fluid through the body. With this view I disjoined the chain which combined the zinc pole of the battery A, with the copper pole *β* of the battery B, and made to it a silver probe, passing through a glass tube, by which it was insulated (Tab. I. e.) I then conducted another silver probe from the zinc pole *α* of the second battery through the vessel *α α* and applied it to the eye; the hand, however, was brought in contact with the copper pole b, by means of the vessel *β β*, and thus the Galvanic chain was shut. My expectations were by no means disappointed, as the effect of the Galvanic agens was considerably increased, the lightning became stronger, and the muscular contractions more violent, in such a degree, that I found myself obliged to diminish the number of the shocks from two hundred to one hundred or one hundred and fifty, and of those that were immediately applied to the eye, from twenty-four to 12 or 15. I applied, at the same time, the silver probe to the septum narium, sometimes higher up, sometimes lower down, while I shut the chain in the above manner. This mode of application acted as the strongest sternutotary, and produced a most intense lightning in the eye. The greatest affection of the sight, however, appeared if the patient was not brought in contact with the copper pole of the combined batteries by the hand, but if a probe was immediately conducted from this pole to the head, eyes, or the septum narium. But as Mr. Ritter has discovered, that while the state of light in one one eye produced by the negative pole is increafed, that at the pofitive pole is diminifhed, which I alfo fuppofed to take place in this manner of making the experiment, I fcrupled to bring the patient frequently within the Galvanic chain in that manner. This increafed action of the Galvanifm being continued for above a fortnight, twice a day, the patient feemed to make a more rapid progrefs towards recovery than before; and on the 29th of June, (I had begun to treat the patient from the 11th of May) the patient was able to perceive light in the eye, when the Galvanic chain continued to be fhut, in confequence of the lightning produced at the zinc pole. This fenfation daily increafed, and he perceived it alfo at the pofitive pole: The patient found, at the fame time, to his utmoft fatisfaction, that he could diftinguifh the out-lines of the objects, which he had not been capable of before. About this time, when the patient conceived the greateft hopes of recovery, I was prevented from continuing the cure, being obliged to leave the place where the patient refided; but I am in hopes, that by a continued ufe of Galvanifm he will be enabled to diftinguifh the objects fufficiently, fhould the obftinacy of his complaint not allow a complete recovery of his fight.

*Case II.* A country girl, 20 years of age, of a bloated habit, had been affected for above a twelvemonth with a dimnefs of fight, which permitted her to diftinguifh only large objects, and the white and black colour, and which made every thing appear as if obfcured by clouds. This complaint feemed to have arifen from a retention of the menfes, originating in a direct debility of the fyftem; and from the fame fource are to be derived the epileptic fits which had from time to time attacked her; after the ceffation of which, the weaknefs of fight had begun and increafed to the degree in which it was at prefent found. She had been treated with the moft efficacious remedies, but without much fuccefs, till I determined to try what the application of Galvanifm might do in this cafe. The apparatus was accordingly begun to be ufed on the 12th of May, after the fame manner as in the Cafe before-mentioned, but not in fuch a degree as had been there required; it fhewed, however, a quicker effect than in the former cafe, and the patient perceived, on the 16th of May, (immediately after the application of Galvanifm,) fome light in the eye, which continued for a few moments. Meanwhile the patient had fymptoms of menftruation, which I endeavoured to fupport by medicines, in order to produce a full effect, but without the expected fuccefs. The fight of the patient, however, improved daily, fo that fhe could fome days after diftinguifh the lighter fhades of an engraved portrait at one ftep diftance, tance, and a few days after the hand and fingers on it, and even the letter-prefs under it. On the 12th of June, the figns of menftruation returned, but they could not by any means be brought to flow. She began now to diftinguifh colours, and at firft the dark ones; eight days after the other colours; and, on the 28th of June, fhe was able to diftinguifh common letter-prefs. On the 2d of July, fhe was able to knit and to work with her needle, and eafily diftinguifhed the phyfiognomy of the perfons that were about her. As the figns of menftruation appeared again from time to time, the patient was ordered to take one night, *Pilul. balsamic. Hoffm.* ℈j. *Extract. taxi gr.* ij. with a tea of *flor. Arnic.* and *chamomill. Roman* but this had not the intended effect. About noon the next day, the Galvanifm being applied to her as ufual, the menfes appeared half an hour after, and continued for three days, fhortly after the application of Galvanifm; which is the more remarkable, as a topical irritation of the genitals, by conducting one pole to the labia pudendi, and the other into the vagina, had not before been capable of producing a fimilar effect.

*Case III.* A woman, aged 28, of a lean habit and yellow complexion, had been brought to bed in December, 1800, but fince that time the right fide, right arm and leg, became quite paralytic, the pulfation of that fide was very weak, and the animal heat confiderably diminifhed; the tongue was likewife palfied, fpeech and tafte almoft entirely abolifhed. No fever, however, and all other functions in juft order. She was ordered to take belladonna with opium, which, being ufed fome weeks, raifed the pulfe a little; but as no other ftep towards recovery was perceived, recourfe was had to electricity. After it had been applied for three weeks the patient received fome motion in the arm, which favourable fymptom however difappeared from time to time, without any evident caufe. But though the efficacy of electricity was again fupported by the internal ufe of belladonna, no great progrefs in the cure could be perceived. On the 11th of May, I proceeded to the application of Galvanifm, after I had previoufly ordered the former remedies to be difcontinued. In the beginning I brought the patient only by the hands within the Galvanic chain, which produced, by degrees, violent contractions in the paralytic arm; but, a few days after, I conducted the copper pole immediately to the tongue, by means of an infulated probe, which caufed vehement contractions and an alkaline tafte, together with an increafed fecretion of faliva. The copper pole was alfo alternately conducted to the region of the nervus vagus, and to the pharynx and larynx. The convulfions in the difeafed arm appeared more violent at the zinc pole, when the copper pole was conducted ducted in the above manner, than if the conductor was merely applied to the left arm. After having thus continued the application of Galvanifm, feveral favourable fymptoms appeared; fhe could move the paralytic arm, the pulfe and animal heat of which were confiderably reftored, fo as to become nearly the fame as in the found arm, and fhe found herfelf more eafy. Eight days after I had increafed the ftrength of the battery, the patient could, though with fome difficulty, raife the arm to the head, and at the fame time fhe made fome efforts to fpeak; but it was not till fome days after that fhe could utter a few words clearly and diftinctly. The progrefs which fhe made in the faculty of fpeech by no means kept pace with the other fymptoms of recovery, though I conducted the Galvanifm to the region of the larynx, after I had previoufly excoriated it by means of a blifter. The patient, however, could now lift and ftretch the hand towards the head; pulfe and animal heat were the fame in both arms, tafte was likewife reftored, alfo the affected leg was in a better ftate, and fhe began to walk pretty fteadily. The dullnefs in which fhe had been all the time feemed to have difappeared, and there was no doubt that the perfect recovery of motion and fenfation in the affected organs, would be attained by the continued application of the Galvanic ftimulus.

*Case IV.* A man, aged 50, who two years ago repulfed an itchy eruption by the imprudent ufe of aftringents, had ever fince that time been afflicted with a chronic afthma, and an arthritic affection in the right fhoulder, which hindered him from moving that arm, and it always caufed him much pain when he attempted it. The patient having ufed the common remedies without much avail, was galvanifed from a battery of 70 ftrata. After having ordered the patient to immerfe the hands into two bowls of falt water, I conducted the copper pole into the veffel in which the left hand was immerfed, while I fhut the Galvanic chain, by plunging and taking out alternately an infulated probe in the veffel into which the hand of the difeafed arm was put. The patient perceived, at each time, a very fenfible fhock, and the flexores digitorum of the hand, at the zinc pole, contracted themfelves; while, in the other hand, the extenfors were put in action, fo that one hand fhut itfelf, and the other opened itfelf, each time the Galvanic chain was formed. The next morning, after the application of Galvanifm, the patient thought himfelf greatly relieved, the pains had almoft entirely ceafed, and motion was pretty well reftored. On the evening of the fame day, however, an oppreffion on the breaft, with a dry cough, fupervened, which ceafed entirely the fame night, when the itchy eruption at the feet was perceived.

A few days after the application of Galvanifm had been continued, the itch difappeared again, while the afthmatic complaints returned; the patient, however, could perfectly move his arm. Having the next day applied the Galvanifm, from a frefh constructed battery of 80 ftrata, the eruption broke out again, which made the complaints in the breaft entirely difappear. Some weeks after the eruption difappeared without any remedy; the patient remained free of any complaint, and was perfectly recovered. During this application of Galvanifm the patient thought his fight, which was naturally weak, to have been confiderably ftrengthened.

*Case V.* A man, aged 43, had been afflicted for above five years with the moft violent epilepfy, which had brought on him fuch a debility that twelve grains of camphor, twice a day, had almoft no effect in exciting him. He had ufed the moft efficacious remedies without experiencing much effect, and the paroxyfms returned once, twice, and fometimes three times a week, fhortly after midnight; the patient was, at the fame time, frequently vexed in day-time with fpaftic affections of the extremities and face. As I had reafon to expect in this cafe fome fuccefs from the application of Galvanifm, I difcontinued all other remedies, and began galvanifing this patient by bringing him in contact with two batteries, each confifting of 60 ftrata, by means of his hands immerfed in the two bowls of falt water, and by fhutting the chain from without the patient. I rofe by degrees from 40 fhocks, which I applied on him twice a day, to about 200, and let him frequently remain for above five minutes within the chain, which laft mode of application I particularly recommend, as acting without interruption on the fenfes. I had hardly applied the Galvanifm in this manner a few days, when the external fpafms ceafed by degrees; and, after having continued about two months, the epileptic paroxyfms intermitted to once in three weeks, and though they returned afterwards two days, one after another, the paroxyfms generally made an intermiffion of a fortnight; and, what I alfo confidered as a favourable fymptom, it came on in the day-time.

*Case VI.* A labourer, 28 years of age, afflicted for above fifteen years with Epilepfy, was galvanifed every day during three weeks, which had fuch beneficial effects that the paroxyfms, which had before attacked the patient once a week, only recurred once within three weeks; I muft however mention, that the patient ufed the flores zinci at the fame time.

During the laft weeks of my ftay at Jena, where I made the above obfervations, I had an opportunity of ufing Galvanifm in a cafe of deafnefs, originating in a debility of the organ; but though my time did not allow me to wait for the fuccefs cefs of this cure, I fhall take the liberty of communicating my method of applying the Galvanifm in this and fimilar cafes. It being particularly requifite to conduct the Galvanic agent into the internal ear, to the nerve and membrana tympani, I paffed a thin filver probe through a glafs tube which was bent in fire according to the form of the ear, as reprefented in Tab. II. fig. 2. It is hung to the ear. One end of the probe (d) is made fufficiently long to be applied to the membrana tympani, and the other end (a) has a hook, to which the conducting chain of the battery can be faftened. The conductor may be alfo infulated by filk, and bent in the above manner. After each ear had been provided with fuch a conductor, I faftened the conducting chain of the copper pole of the battery, confifting at firft of fixteen ftrata only, to the hook of the conductor, Tab. II. fig. 1, a, at the ear that was leaft affected, while I fhut the chain by means of an infulated probe (b) applied to the hook of the conductor of the other ear (c). When the Galvanic chain was fhut in this way, the patient fuffered a fevere fhock, and thought he heard the found of a large bell, which continued as long as the chain was fhut; and always a tingling of the ear remained for feveral hours. I have likewife tried to conduct one pole to the tuba Euftachii, which, though extremely troublefome and painful to the patient, is promifing of much good. The beft way of applying the probe is through the nofe; on which account, however, we fhould infulate it properly, in order to prevent any difagreeable fternutation. This may be effected by covering the probe as far as it touches the fides of the nofe with refina elaftica previoufly diffolved in naphta or ether. Having introduced the probe into the tuba Euftachii by means of the *tour maitre*, I combined the hook with the conductor of the copper pole, and fhut the Galvanic chain by an infulated probe, conducted from the zinc pole. I have added to Tab. I. fig. C, the Galvanic collar invented by Dr. Grapengieffer for cafes of Aphonia: It confifts of a ftrap with a buckle, to which are faftened, by means of buttons and button-holes, a zinc plate and a copper or filver plate. Both plates are furnifhed with a hook, by means of which they are combined with the battery.

*Description of the Galvanometer*, Tab. II. fig. 3 and 4.— Mr. Ritter hung in a glafs receiver a piece of gold leaf, and introduced a wire through a hole in the fide of it; when a connection was made between the wire and the gold leaf, the oppofite poles attracted each other. This experiment was feveral times repeated, and according as the effect of the battery was ftronger or weaker the attraction took place at a greater or fmaller diftance. It was evident therefore, that a fcale of the Galvanic action might be conftructed by obferving the diftance in which the two bodies attracted each other. For this purpofe it was required to take notice of the fmalleft variation of the gold leaf from the vertical line, and at the fame time accurately obferve the diftance.

Fig. 4, Tab. II. is a fketch of the inftrument. A, the fides of a glafs cylinder upon which is a cover of metal, bb; the brafs wire, o, paffes through a hole, and is faftened by the fprings at cc. It may be gently moved backward and forward by means of the micrometer fcrew, dd, the nature of which will be eafily underftood, vide ee, f, kk, ii, &c. EEE is a flat fcale, moveable in the cafe GG, by means of a wheel and teeth. It bears the globe D, which is to attract the gold leaf, faftened to a piece of brafs, BB, with a little gum; this piece of brafs is faftened in the wire, o, by means of a fcrew, fo that it may be taken out. The cafe GG, in which the fcale EEE moves, is cemented to the cylinder with a round plate; here the cafe is larger and contains the wheel, into which the teeth of the fcale work; the axis of this wheel is of glafs, in order to infulate it. The cafe is open, in order to be able to fee the divifions of the fcale, which meafures the diftance between the globe D, and the gold leaf. On the cafe at F is a fubdivifion for the purpofe of determining the lines in the fcale. A ftand with fcrews fupports the whole. From the greater or lefs diftance of the globe from the gold leaf, which is pointed out by the fcale, we are enabled to determine the ftrength of the battery.

## Figures and Tables

**Fig 1 Fig. 2. Fig. 3. Fig. 4. Fig 5. Tab. 2. f1:**